# Editorial: Etiopathogenesis of Systemic Sclerosis: An Update 

**DOI:** 10.3389/fimmu.2021.663381

**Published:** 2021-03-22

**Authors:** Giuseppina Stifano, Raffaele De Palma

**Affiliations:** ^1^ Arthritis Center, Boston University School of Medicine, Boston, MA, United States; ^2^ Dipartimento di Medicina Interna e Specialità Mediche (DIMI), University of Genova, Genova, Italy; ^3^ Clinical Immunology, IRCCS Ospedale Policlinico San Martino-IST-Genova, Genova, Italy

**Keywords:** systemic sclerosis (scleroderma), autoimmunity, vascular injury, fibrosis, immune response

Systemic Sclerosis (SSc) or Scleroderma is a complex and puzzling disease having an incidence of 1–2 cases per 100,000. In SSc, inflammation leads to organ failure due to severe fibrosis of the skin and internal organs. At late stages, this disease is characterized by a profound decline in the quality of life and premature death (1). The Research Topic “Etiopathogenesis of Systemic Sclerosis: An Update” is aiming to explore three major features of SSc: vascular injury, fibrosis, and immune dysregulation. Here, we summarize the novel insights reported in this Topic with a list of references to further exploit the given issues. Apostolidis et al. identify genes linked to vascular injury in SSc by scRNA-seq. Among several genes, they found Apelin Receptor (APLNR) and Heparan Sulfate Proteoglycan 2 (HSPG2), not yet associated to SSc pathogenesis but of great interest as they have been reported to sustain vascular dysfunction and fibrosis in different settings (2). These data provide the ground to characterize new biomarkers of vascular injury and, possibly, therapeutic targets. Svegliati et al. define the role of the Anti- platelet- derived growth factor (PDGF) autoantibodies in vascular injury. The authors analyze the expression of distinct functional markers of smooth muscle cells (SMC) from human pulmonary arteries (HPASMC) exposed *in vitro* to anti-PDGFR autoantibodies from SSc patients. They show that PDGFR autoantibodies activate SMC and may contribute to the development of SSc vascular lesions, therefore suggesting that a downregulation of B cell response could modify disease development (3). Napolitano et al. show that *N*-formyl peptide receptors (FPRs) can induce Reactive Oxygen Species (ROS) generation in fibroblasts through the interaction with the urokinase-type plasminogen activator/uPA receptor (uPA/uPAR) system, activation of the nicotinamide adenine dinucleotide phosphate (NADPH) oxidase and alteration of the redox state observed in SSc. Napolitano*’s* data indicate novel therapeutic strategies in SSc, through the use of small molecules to impair FPRs functional interaction with uPAR and their signal (4). Dolcino et al. suggest the presence of modulated genes and miRNAs playing a predisposing role in the development of malignancies in SSc. Genetic and epigenetic features shared by SSc and cancer shed new light on the pathogenesis of the disease and support the idea that immune activation against a tumor may have a central role in the initiation and progression of SSc, as also indicated by the presence or development of malignancies associated with particular autoantibodies (5). Xiong et al. show that in a murine model of SSc, Sclerodermatous Graft Versus Host Disease (sclGvHD), daily stretching produced a measurable beneficial on reducing skin thickness and improved mobility during the fibrotic phase of the model. Of note, stretching reduced mRNA expression of C-C Motif Chemokine Ligand-2 (CCL2) and a disintegrin and metalloproteinase domain-8 (ADAM8) which are important inflammatory mediators associated with the sclGvHD model and upregulated in SSc skin. B cells appear to have an important role in SSc pathophysiology beyond the classical production of autoantibodies. Data on B cells subsets distribution and functional properties in animal models of SSc are scarce. Moreover, no study has ever considered a possible variation in B cell involvement during the course of SSc. This is of peculiar importance since several works have suggested that B cell-targeted therapeutic strategies may have different effects whether they are started at an early or late stage of the disease. To address these issues (6), Sanges et al. use a novel murine model of SSc in which daily intradermal injections of hypochlorous acid (HOCl) induce a systemic fibrosis. The authors study the modifications in B cell homeostasis. Phenotypic analyses show an early expansion of the mature naïve subset, decrease in plasmablasts, and memory B cells. Functional analyses reveal B-cell overproduction of pro-inflammatory cytokines (Interleukin 6 and CCL3) and an impairment of their anti-inflammatory capacities (decreased production of IL-10 and Transforming growth factor-β and reduced levels of B-regs) at the early inflammatory stage, followed by an overproduction of pro-fibrotic cytokines (TGF-β and IL-6) at the late fibrotic stage. This work reports, for the first time in an SSc animal model, the existence of B cell dysfunctions similar to those observed in SSc patients. It is of note that these anomalies vary over the course of the disease and may contribute to the inflammatory and fibrotic events observed in SSc. This makes the HOCl mouse a relevant experimental model for the study of B cells, and especially B cell- targeted therapies, in SSc (7). SSc is also characterized by alterations of the normal function of T cell, in particular an unbalanced ratio between the effector and regulatory arms of the immune system. Negrini et al. report that CD8+ T-regulatory (T-reg) subsets display functional defects in SSc patients (8), suggesting an impairment of maturation processes affecting CD8+ T-reg cells in SSc patients. This impairment of maturation involves phenotypic alterations that are mainly characterized by a deficient CD39 upregulation and a lack of down-modulation of the CD127 molecule. These data may shed a new light in the dysfunction of immune response underlying SSc. Vettori et al. investigate the dynamics and the function of T cell–fibroblast interaction in SSc, using an experimental co-culture setting, highlighting the role of IL-17A. IL-17 has been proposed to have a central role in SSc (9). The authors show that T cell–fibroblast co-cultures overexpress IL17A and IL17RA, co-cultured fibroblasts also upregulate IL-17A targets while two key effectors of the TGF-β signaling, *TGFBR2* and *SMAD3*, are downregulated. Simultaneous α-IL-17RA mAb treatment restore ProCollagen I levels and reduce fibroblast apoptosis in IL-17A-stimulated co-cultures. Long et al. review the mechanisms regulating ubiquitination in SSc and explore potential anti-fibrosis drugs (10). Considering the central role of TGF-β signaling WNT/β-catenin signaling and STAT3 in SSc, the use of ubiquitin–proteasome system (UPS) inhibitors to selectively disrupt the formation of receptor or co-receptor complexes or block intracellular signaling may yield advances in the development of urgently needed treatments.

## Author Contributions

The two authors share the supervision of the papers included in the Research Topics and the expressed view. All authors contributed to the article and approved the submitted version.

## Conflict of Interest

The authors declare that the research was conducted in the absence of any commercial or financial relationships that could be construed as a potential conflict of interest.
